# An adult and pediatric size‐based contrast administration reduction phantom study for single and dual‐energy CT through preservation of contrast‐to‐noise ratio

**DOI:** 10.1002/acm2.14340

**Published:** 2024-04-11

**Authors:** Jia Wang, Xinhui Duan, Usman Mahmood, Sarah Eva McKenney, Samuel Loren Brady

**Affiliations:** ^1^ Department of Environmental Health & Safety Stanford University Stanford California USA; ^2^ Department of Radiology UT Southwestern Medical Center Dallas Texas USA; ^3^ Department of Medical Physics Memorial Sloan Kettering Cancer Center New York USA; ^4^ Department of Radiology University of California, Davis Medical Center Sacramento California USA; ^5^ Department of Radiology Cincinnati Children's Hospital Medical Center University of Cincinnati Cincinnati Ohio USA

**Keywords:** dual‐energy CT, iodinated contrast media, iterative reconstruction, virtual monochromatic images

## Abstract

**Background:**

Global shortages of iodinated contrast media (ICM) during COVID‐19 pandemic forced the imaging community to use ICM more strategically in CT exams.

**Purpose:**

The purpose of this work is to provide a quantitative framework for preserving iodine CNR while reducing ICM dosage by either lowering kV in single‐energy CT (SECT) or using lower energy virtual monochromatic images (VMI) from dual‐energy CT (DECT) in a phantom study.

**Materials and Methods:**

In SECT study, phantoms with effective diameters of 9.7, 15.9, 21.1, and 28.5 cm were scanned on SECT scanners of two different manufacturers at a range of tube voltages. Statistical based iterative reconstruction and deep learning reconstruction were used. In DECT study, phantoms with effective diameters of 20, 29.5, 34.6, and 39.7 cm were scanned on DECT scanners from three different manufacturers. VMIs were created from 40 to 140 keV. ICM reduction by lowering kV levels for SECT or switching from SECT to DECT was calculated based on the linear relationship between iodine CNR and its concentration under different scanning conditions.

**Results:**

On SECT scanner A, while matching CNR at 120 kV, ICM reductions of 21%, 58%, and 72% were achieved at 100, 80, and 70 kV, respectively. On SECT scanner B, 27% and 80% ICM reduction was obtained at 80 and 100 kV. On the Fast‐kV switch DECT, with CNR matched at 120 kV, ICM reductions were 35%, 30%, 23%, and 15% with VMIs at 40, 50, 60, and 68 keV, respectively. On the dual‐source DECT, ICM reductions were 52%, 48%, 42%, 33%, and 22% with VMIs at 40, 50, 60, 70, and 80 keV. On the dual‐layer DECT, ICM reductions were 74%, 62%, 45%, and 22% with VMIs at 40, 50, 60, and 70 keV.

**Conclusions:**

Our work provided a quantitative baseline for other institutions to further optimize their scanning protocols to reduce the use of ICM.

## 1 INTRODUCTION

Attempts to reduce iodinated contrast medium (ICM) has historically been investigated as a means to reduce acute kidney injury^1^; however, recent global shortages of ICM forced the imaging community to ration supply and use ICM with computed tomography (CT) equipment more strategically.^2^, ^3^, ^4^, ^5^ For example, Davenport et al. suggest that indication, weight, and kV‐based strategies can reduce contrast consumption by 83% in adult patients.^6^ Although the proposed strategies may help conserve ICM, the risk of inadvertently generating suboptimal or non‐diagnostic images increases since several variables related to the diagnostic task, including the patient's weight, age, and the osmolarity of the contrast media, must be considered^7^; additionally, the lower threshold for ICM administration before a CT examination becomes non‐diagnostic is not well defined. Consequently, many ICM dose reduction efforts remain an iterative, bootstrapping process that may not consider using the full extent of features available on any given CT scanner.

Two technological developments that considerably shifted ICM‐enhanced CT imaging include reduced tube potential, that is, low kV imaging with high‐capacity x‐ray tubes using single‐energy CT (SECT), and dual‐energy CT (DECT). Historically, reducing tube potential enhanced the contrast of iodinated tissues, but caused an increase in image noise because tube current was limited by the generator power capacity. As a result, improved delineation of adjacent tissues associated with reduced kV imaging was limited to smaller patients. With modern noise reduction schemes, such as iterative or deep learning reconstruction and increased x‐ray tube output, consistent contrast‐to‐noise ratio (CNR) across kV stations is achievable. Similarly, with DECT, the relative intensity of tissues perfused with iodine can be increased by reconstructing lower energy virtual monochromatic images (VMI).^8^

Although current best practices for modifying CT protocols exist,^3^, ^9^, ^10^, ^11^, ^12^, ^13^ the sudden onset of the ICM shortage required medical practitioners to rapidly implement new CT scan protocols informed by literature resources or institutional experiences. Unfortunately, vendor‐specific DECT solutions differ considerably in their implementation and transferring knowledge between vendors is complicated. Moreover, the available literature on the impact of reduced contrast dosages on CNR is limited to single‐vendor solutions, to only adult patients, or multi‐vendor options from a single site.

In this study, we addressed the limitations and filled the gaps with a multi‐vendor study that aimed to create a quantitative framework for preserving CNR while reducing ICM dosages for adult and pediatric patients. We evaluated contrast conspicuity and CNR using physical semi‐anthropomorphic phantoms that simulated the attenuation of adult and pediatric patients and included four CT vendors with SECT and DECT scanning capability. The phantoms consisted of inserts with different concentrations of iodinated contrast and were scanned with a range of settings typically observed in adult and pediatric clinics. CT attenuation and CNR values of the iodinated contrast inserts were compared to identify the achievable contrast dose reduction for a given scanner, patient size, SECT reconstruction type, and DECT versus SECT.

## 2 MATERIALS AND METHODS

In this study, two different sets of semi‐anthropomorphic abdomen phantoms were imaged using technology from four different CT manufacturers to investigate ICM reduction under SECT and DECT conditions; acquisition parameters on the different scanners reflect different institutional preferences.

### 2.1 ICM reduction at low SECT kV using advanced CT reconstruction techniques

#### 2.1.1 Phantoms

**FIGURE 1:**
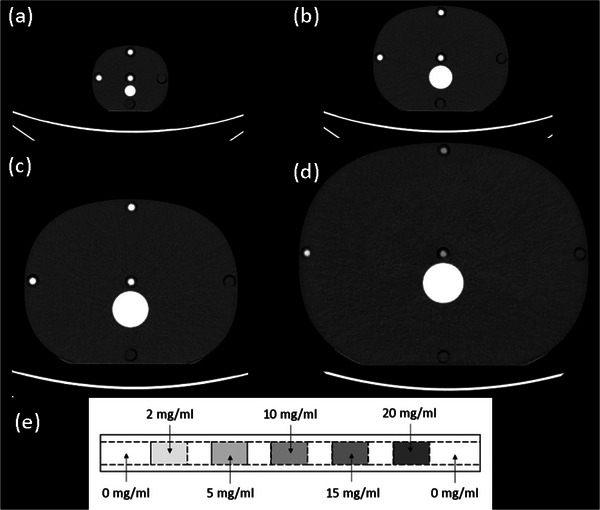
Four tissue equivalent abdomen phantoms, (a) newborn—9.7 cm, (b) 5 YO—15.9 cm, (c) 15 YO—21.1 cm, (d) and medium adult—28.5 cm, were imaged with (e) iodine contrast insert rods that contained contrast concentrations from 2 to 20 mg/mL. The rods were placed at the periphery and at the center of the phantom, and each rod spanned the entire 15 cm z‐axis length of each phantom. YO, year‐old.

Four tissue equivalent physical anthropomorphic abdomen phantoms (Model 007TE CIRS, Norfolk VA) were imaged with contrast insert rods made to emulate iodine contrast enhancement at different concentrations. The phantom's effective diameters were: newborn—9.7 cm, 5 year‐old (YO)—15.9 cm, 15 YO—21.1 cm, and medium adult—28.5, (Figure 1(a–d)); z‐axis length was 15 cm for all phantoms. Each phantom had five holes, arranged with four around the periphery and one at the center. Three customized tissue equivalent rods (13.1 mm diameter by 150 mm long) were each designed with sections, approximately 2 cm long, containing contrast ratios of: 2, 5, 10, 15, and 20 mg/mL (Figure 1(e)).

#### 2.1.2 Acquisition parameters

Each phantom, with contrast rod in situ, was scanned using a SECT scanner. Phantom imaging and reconstruction parameters for a GE Revolution Apex (GE Healthcare, Waukesha, WI, USA), referred to as SECT scanner A, and a Toshiba Aquilion One Genesis edition (Canon Medical, Otawara, Japan), referred to as SECT scanner B, are listed in Table 1. CTDI_vol_ was matched across both SECT systems for each phantom size respectively. Each phantom acquisition was reconstructed using ASiR‐V (Standard kernel, 50% setting, GE Healthcare) and statistical based iterative reconstruction (SBIR), AIDR3D (FC‐18H kernel, standard setting, Canon Medical). All images were then retrospectively reconstructed using deep learning reconstruction (DLR) algorithms: TrueFidelity (Medium setting, GE Healthcare) and AICE (Standard Body setting, Canon Medical).

**TABLE 1:** Scanning and reconstruction parameters used in the low SECT kV study.

CT system	GE Revolution Apex	Canon Aquilion One Genesis
Reference title	SECT scanner A	SECT scanner B
**Scanning parameters**		
Tube voltage of single energy CT scan (kV)	70, 80, 100, 120, 140	80, 100, 120, 135
Beam collimation (mm)	40	40
Pitch	0.99	0.813
Rotation time (s)	0.5	0.5
**Reconstruction parameters**		
Image thickness (mm) & reconstruction interval	5	5
Reconstruction kernel	Standard	FC‐18H
Reconstruction algorithm	ASiR‐V 50% & TrueFidelity	AIDR3D Standard & AiCE

### 2.2 ICM reduction using DECT versus SECT scanners

#### 2.2.1 Phantoms

**FIGURE 2:**
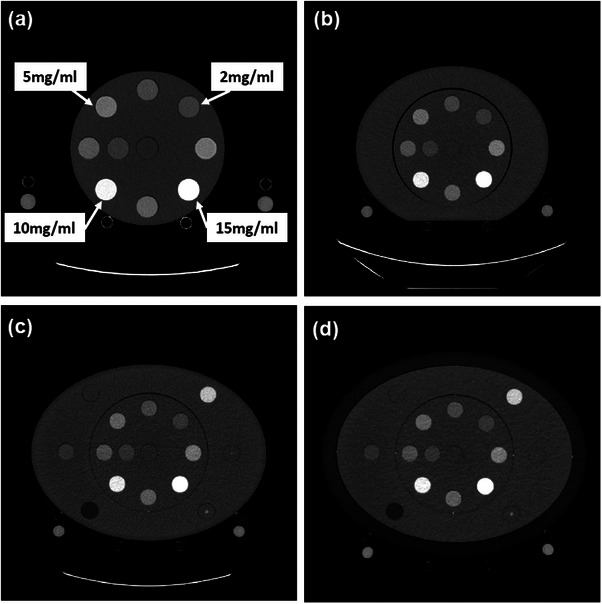
CT image of phantoms with iodine inserts at four concentrations (2, 5, 10, and 15 mg/mL). Phantoms of four different sizes were studied: (a) Small Adult—20 cm, (b) Medium Adult—29.5 cm, (c) Large Adult—34.6 cm, and (d) Extra Large Adult—39.7 cm.

A cylinder phantom with diameter of 20 cm was used to house four iodine inserts with concentrations of 2, 5, 10 and 15 mg/mL (Multi‐Energy CT Phantom, Sun Nuclear Inc., Middleton, WI, USA). The diameter of each iodine insert was 28.5 mm. The z‐axis length of the phantom was 16.5 cm. To mimic patients of different body size, the cylindrical phantom was enclosed by elliptical annular rings. Phantom effective diameter's were: Small Adult—20 cm, Medium Adult—29.5 cm, Large Adult—34.6 cm, and Extra Large Adult—39.7 cm (Figure 2(a–d)). The cylindrical body of the phantom, in Figure 2(a), and elliptical annular rings in Figure 2(b) and (c) are made of solid water material (Sun Nuclear Inc., Middleton, WI, USA), and the outer elliptical annular ring in Figure 2(d) is custom‐made using polyethylene with CT number equivalent to fat. Other sensitometry inserts observed in Figure 2 were not used in this study.

#### 2.2.2 Acquisition parameters

Adult phantoms of four different sizes were scanned on three clinical DECT enabled scanners: 1) fast‐kV switching CT (Revolution, GE Healthcare), 2) dual‐source CT (SOMATOM FORCE, Siemens Healthineers, Erlangen, Germany), and 3) dual‐layer detector CT (iQon, Philips Healthcare, Amsterdam, Netherlands). On each DECT scanner, the acquisitions per phantom included: SECT scans at 100 and 120 kV along with DECT scans. The scanning and reconstruction parameters displayed in Table 2 for the DECT systems were based on institution's routine adult body CT protocols. Automatic exposure control (AEC) was enabled for the Siemens and Philips DECT scans. Fixed mA was used for the GE DECT scanner. CTDI_vol_ was matched across all three DECT systems for each phantom size, respectively. VMI's were created at energy levels ranging from 40 to 80 keV.

**TABLE 2:** Scanning and reconstruction parameters used in the DECT versus SECT study.

CT system	GE revolution	Siemens force	Philips iQon
Reference title	Fast kV switching scanner	Dual source scanner	Dual layer scanner
**Scanning parameters**			
Tube voltage of single energy CT scan (kV)	100, 120	100, 120	100, 120
Energy pair of dual energy CT scan (kV)	80/140	80/150 with (Sn filter)	120
Beam collimation (mm)	80	57.6	40
Pitch	0.984	1	Auto
Rotation time (s)	0.8	0.5	Auto
**Reconstruction parameters**			
Image thickness (mm) & reconstruction interval	2.5	2.5	2.5
Reconstruction kernel	Standard	Qr40	B
Reconstruction algorithm	ASiR‐V 50%	ADMIRE Level 3	iDose Level 3

### 2.3 Data analysis

CNR was calculated for all phantoms in this study by placing a circular region of interest (ROI) within the inner diameter of the contrast inserts, and in the surrounding phantom background using an automatic method (MATLAB, Mathworks, Natick MA). For each phantom size scanned, CNR versus ICM was plotted. The plots of CNR were fitted using a linear function for each energy level:

(1) $$SE-CNR_{kV,size}=\alpha_{kV,size}\times Conc_{kV,size}$$

(2) $$DE-CNR_{keV,size}=\alpha_{keV,size}\times Conc_{keV,size}$$

where $SE-CNR_{kV,size}$ and $DE-CNR_{keV,size}$ represent the CNR of iodine inserts on single‐energy (SE) and dual‐energy (DE) images as a function of either SE kV and VMI, that is, keV reconstruction setting, and for each phantom size shown in Figures 1 and 2. $Conc_{kV,size}$ and $Conc_{keV,size}$ are concentrations of different iodine inserts, and $\alpha_{kV,size}$ and $\alpha_{keV,size}$ represent the slope of the fitted linear function. The amount of iodine reduction by changing kV levels for SECT (from *kV_1_* to *kV_2_) or* switching from SECT to DECT (from *kV* to *keV)* can be calculated:

(3) $$Conc_{kV2,size}=\left(\frac{\alpha_{kV1,size}}{\alpha_{kV2,size}}\right)\times Conc_{kV1,size},$$

(4) $$Conc_{keV,size}=\left(\frac{\alpha_{kV,size}}{\alpha_{keV,size}}\right)\times Conc_{kV,size},$$

(5) $$\begin{array}{ccc} Iodine\;reduction\;{\left(\%\right)}_{SECT} & = & 1-\left(\frac{Conc_{kV1,size}}{Conc_{kV2,size}}\right)\\ & = & 1-\left(\frac{\alpha_{kV1,size}}{\alpha_{kV2,size}}\right), \end{array}$$

(6) $$\begin{array}{ccc} Iodine\;reduction{\left(\%\right)}_{DECT} & = & 1-\left(\frac{Conc_{keV,size}}{Conc_{kV,size}}\right)\\ & = & 1-\left(\frac{\alpha_{kV,size}}{\alpha_{keV,size}}\right). \end{array}$$

With the proposed approach, the amount of ICM reduction when switching tube potentials or between SECT and DECT can be calculated for all phantom sizes.

### 2.4 ICM reduction patient study

We illustrate the applicability of the phantom approach by analyzing two patient studies. Institutional review board approval was obtained for this Health Insurance Portability and Accountability Act‐compliant retrospective study. The requirement for informed consent was waived. All patient image data were collected retrospectively.

The full contrast dose scan was performed for both patients in January 2022 and included the administration of 150 mL of ICM (Iohexol 300 mgI/mL, Omnipaque 300, GE Healthcare, Cork, Ireland) at 2.5 mL/s. Both patients received a follow‐up DECT Urogram 4 months after their initial scans (May 2022) as part of routine patient care. Due to the contrast shortage at the time of the follow‐up scans, the contrast volume was decreased by 33% to 100 mL (Iohexol 300 mgI/mL) at 2.5 mL/s.

All scans were acquired on a 64‐slice fast kV switching DECT scanner (Discovery CT750 HD, GE Healthcare) using a GSI‐22 preset, pitch of 0.984, 0.8 s rotation time, slice thickness, and reconstruction interval of 5 mm, 15.02 mGy CTDI_vol_. The VMIs were generated using the gemstone spectral imaging (GSI) MD Analysis software (Advantage Workstation Volume Share 7, GE Healthcare) for the full and reduced ICM dose scans. In this work, VMIs of 70 keV were used as a surrogate of 120 kV single energy images for image quality comparison with VMIs of lower energy levels.

## 3 RESULTS

### 3.1 ICM reduction at low SECT kV using advanced CT reconstruction techniques

**FIGURE 3:**
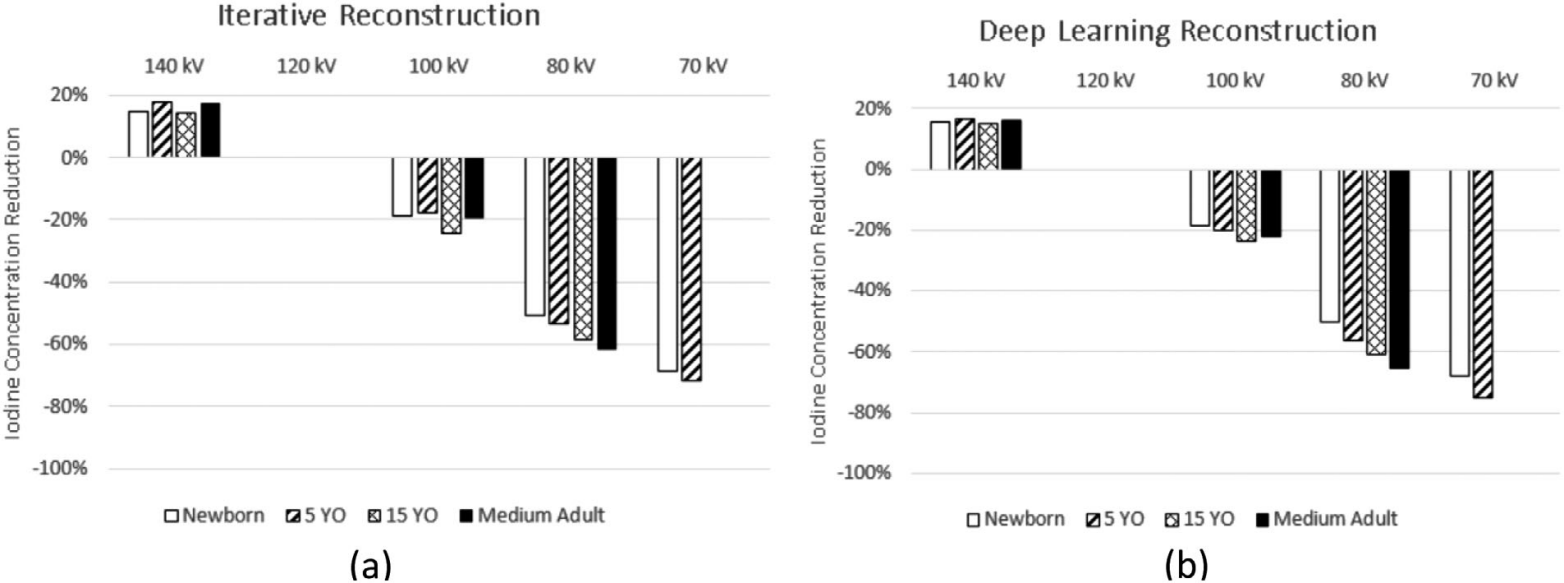
Data acquired on a GE Revolution Apex. Percent iodine concentration reduction is shown as normalized to images of the phantoms acquired at 120 kV. Four phantoms of varying effective diameters were used to measure CNR, namely: newborn—9.7 cm, 5 YO—15.9 cm, 15 YO—21.1 cm, and medium adult—28.5 cm. Iodine reduction was assessed independently for (a) SBIR and (b) deep learning reconstruction algorithms. Note: 70 kV was not feasible for the 15 YO and medium adult phantoms, the mA output was not sufficient to maintain noise, and conversely, CNR properties in the reconstructed image. YO, year‐old.

**FIGURE 4:**
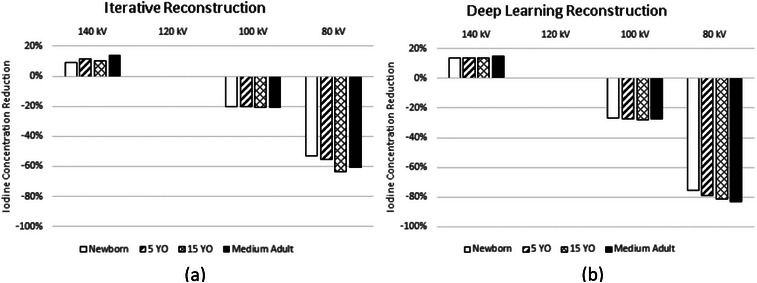
Data acquired on a Canon Aquilion One Genesis. Percent iodine concentration reduction is shown as normalized to images of the phantoms acquired at 120 kV. Four phantoms of varying effective diameters were used to measure CNR, namely: newborn—9.7 cm, 5 YO—15.9 cm, 15 YO—21.1 cm, and medium adult—28.5 cm. Iodine reduction was assessed independently for (a) iterative reconstruction and (b) deep learning reconstruction algorithms. YO, year‐old.

**FIGURE A1:**
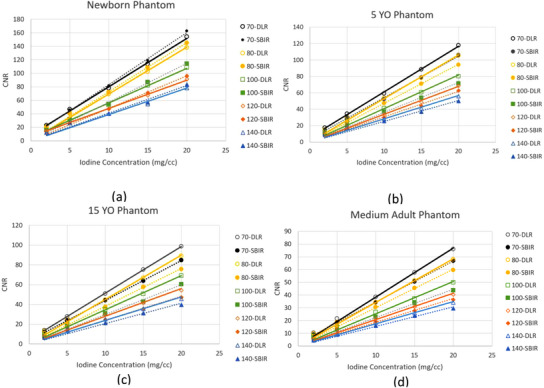
Data acquired on a GE Revolution Apex. CNR calculated for four tissue equivalent abdomen phantoms (a) newborn—9.7 cm, (b) 5 YO—15.9 cm, (c) 15 YO—21.1 cm, (d) and medium adult—28.5 cm, were imaged with contrast insert rods (Figure 1(e)) that contained contrast ratios from 2 to 20 mg/mL. The rods were placed along the periphery and at the center of the phantom. YO, year‐old.

**FIGURE A2:**
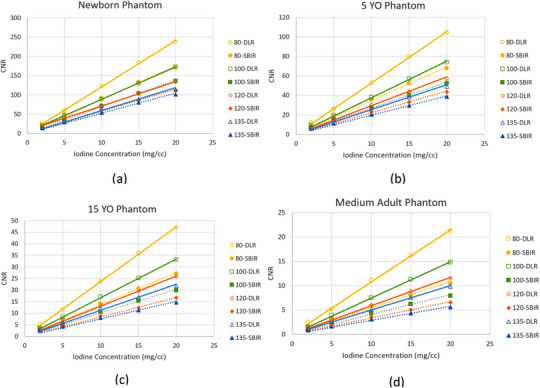
Data acquired on a Canon Aquilion One Genesis. CNR calculated for four tissue equivalent abdomen phantoms (a) newborn—9.7 cm, (b) 5 year‐old (YO)—15.9 cm, (c) 15 YO—21.1 cm, (d) and medium adult—28.5 cm, were imaged with contrast insert rods (Figure 1(e)) that contained contrast ratios from 2 to 20 mg/mL. The rods were placed along the periphery and at the center of the phantom. YO, year‐old.

The rate of CNR change for SECT scanners is linearly correlated with iodine concentration (Figures A1 & A2). Reduction of ICM contrast, for SECT scanners, was calculated per equations (3) and (5), as a function of kV, and is shown for SBIR and DLR for SECT scanner A in Figure 3 and for SECT scanner B in Figure 4. The contrast dose reductions were normalized based on the acquisition techniques acquired using 120 kV. Both Figures show the amount of contrast reduction that may be achieved without a loss of CNR in the phantoms.

For SECT scanner A, average reductions were calculated across all phantom sizes of 20% and 56% at 100 and 80 kV, respectively, and 70% at 70 kV for the two smallest phantoms when reconstructing with SBIR (Figure 3(a)), and 21% and 58% at 100 and 80 kV, respectively, and 72% with 70 kV when reconstructing with DLR (Figure 3(b)). Imaging using 70 kV was not feasible for the 15 YO and medium adult phantoms, the mA output was not sufficient to maintain noise, and conversely, CNR properties in the reconstructed image. On average, for all phantom sizes, images acquired at 140 kV require an increase of 16% of ICM dose to match CNR conditions at 120 kV.

For SECT scanner B, average reductions were calculated across all phantom sizes of 21% and 58% at 100 and 80 kV, respectively, when reconstructing with SBIR (Figure 4(a)), and 27% and 80% when reconstructing with DLR (Figure 4(b)). On average, for all phantom sizes, images acquired at 135 kV require an increase of 13% of ICM dose to match CNR conditions at 120 kV.

### 3.2 ICM reduction using DECT versus SECT scanners

**FIGURE 5:**
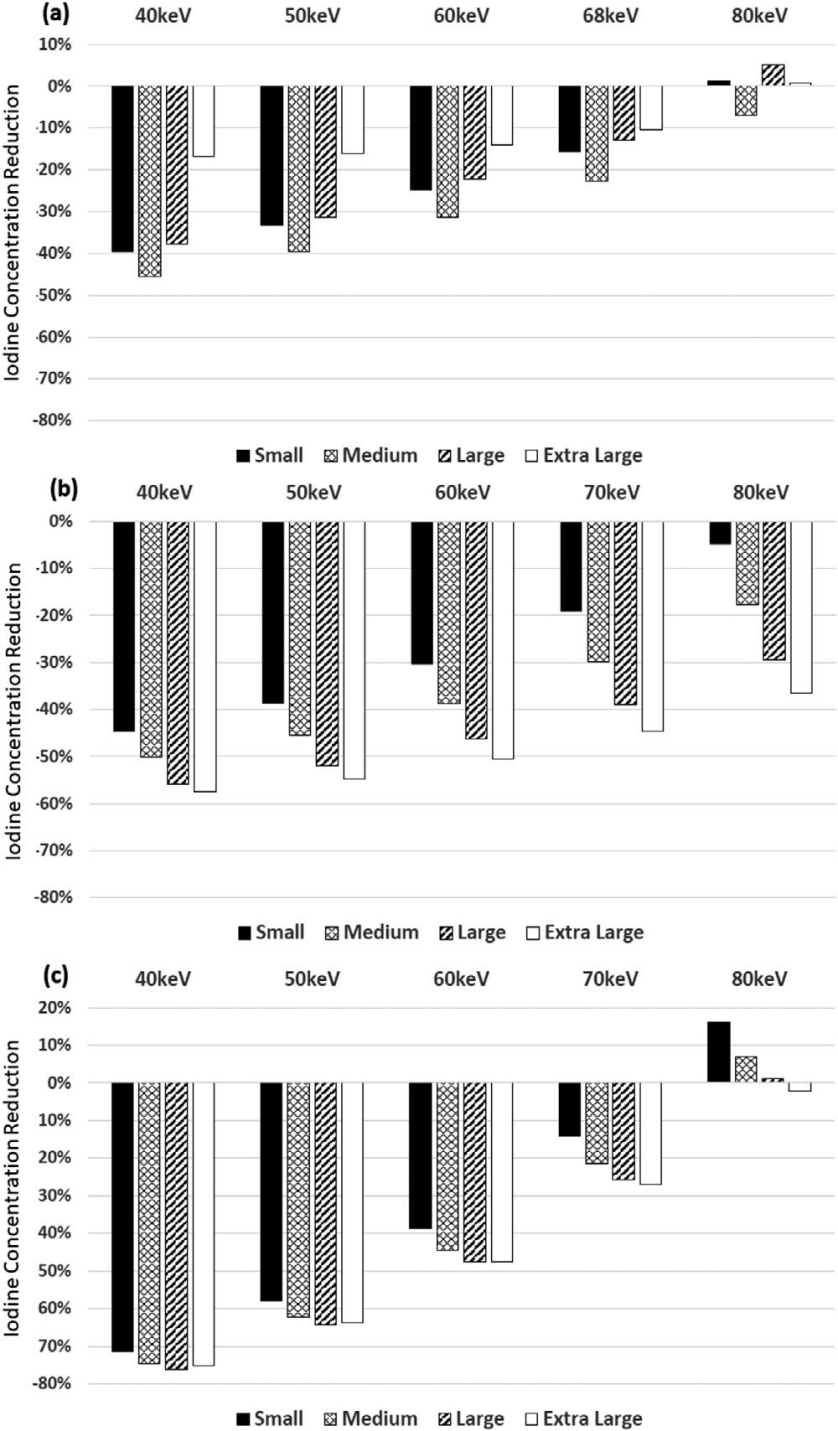
With CNR and dose kept unchanged, the amount of ICM reduction using DECT‐derived VMI at 40, 50, 60, 68/70, and 80 keV, in comparison to single energy 120 kV images, for the four phantom sizes: (a) fast‐kV switching scanner, (b) dual‐source scanner, and (c) dual‐layer scanner. Effective diameters: Small Adult—20 cm, Medium Adult—29.5 cm, Large Adult—34.6 cm, and Extra Large Adult—39.7 cm.

Percentages of ICM reduction were calculated based on Equations (4) and (6) for DECT‐derived VMI energy levels: 40, 50, 60, 68/70, and 80 keV to match CNR conditions at 120 kV. For all three DECT systems, across all four phantom sizes, using lower energy VMI allowed more ICM reduction. For the fast‐kV switching scanner (Figure 5(a)), the average ICM reduction over four phantom sizes were 35%, 30%, 23%, and 15% with 40, 50, 60, and 68 keV images, respectively. Among the four phantom sizes, the highest ICM reduction was found at the medium phantom: 45.6% at 40 keV, 39.7 at 50 keV, 31.4% at 60 keV, and 22.8% at 68 keV. At 80 keV, ICM reduction of 7.1% was only found in the medium phantom, but not in the other three phantoms.

For the dual‐source scanner (Figure 5(b)), the average ICM reduction over four phantom sizes were 52%, 48%,42%, 33%, and 22% with 40, 50, 60, 70, and 80 keV images, respectively. The magnitude of ICM reduction increased as phantom size increased with the highest ICM reduction calculated to be 57.4% at 40 keV, 54.7% at 50 keV, 50.6% at 60 keV, 44.6% at 70 keV, and 36.5% at 80 keV for the extra‐large phantom.

For the dual‐layer scanner (Figure 5(c)), the average ICM reduction over four phantom sizes were 74%, 62%, 45%, and 22% with 40, 50, 60, and 70 keV images, respectively. Similar to the dual‐source scanner, larger ICM reduction was found in larger phantom sizes at all keVs, however the difference of ICM reduction among phantoms is smaller in the dual‐layer scanner.

**FIGURE 6:**
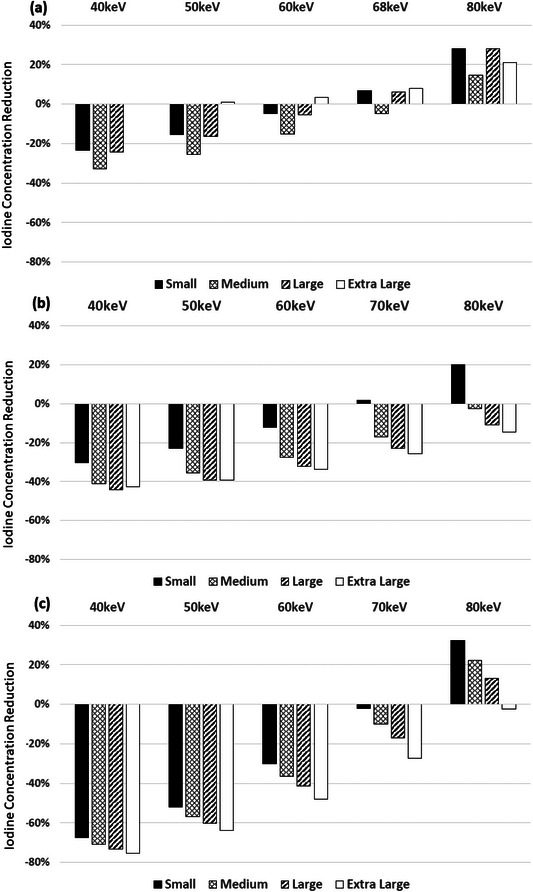
With CNR and radiation dose kept unchanged, the amount of iodine reduction using DECT‐derived VMI at 40, 50, 60, 68/70, and 80 keV, in comparison to single energy 100 kV images, for the four phantom sizes: (a) fast kV‐switching scanner, (b) dual‐source scanner, and (c) dual‐layer scanner. Effective diameters: Small Adult—20 cm, Medium Adult—29.5 cm, Large Adult—34.6 cm, and Extra Large Adult—39.7 cm.

Similarly, comparisons between DECT and 100 kV scans are demonstrated in Figure 6. The magnitude of ICM reduction is less for the same VMI energy and phantom size combination from the 120 kV scan because images of 100 kV had higher ICM CNR initially than those of 120 kV. For the fast‐kV switching scanner, average ICM reduction among four phantoms were 20.1%, 14.2%, and 5.5% at 40, 50, and 60 keV, respectively. For the dual‐source scanner, average ICM reduction were 40%, 34%, 26%, 16%, and 2% at 40, 50, 60, 70, and 80 keV. For the dual‐layer scanner, average ICM reduction were 72%, 58%, 39%, and 14% at 40, 50, 60, and 70 keV.

### 3.3 ICM reduction patient study

**FIGURE 7:**
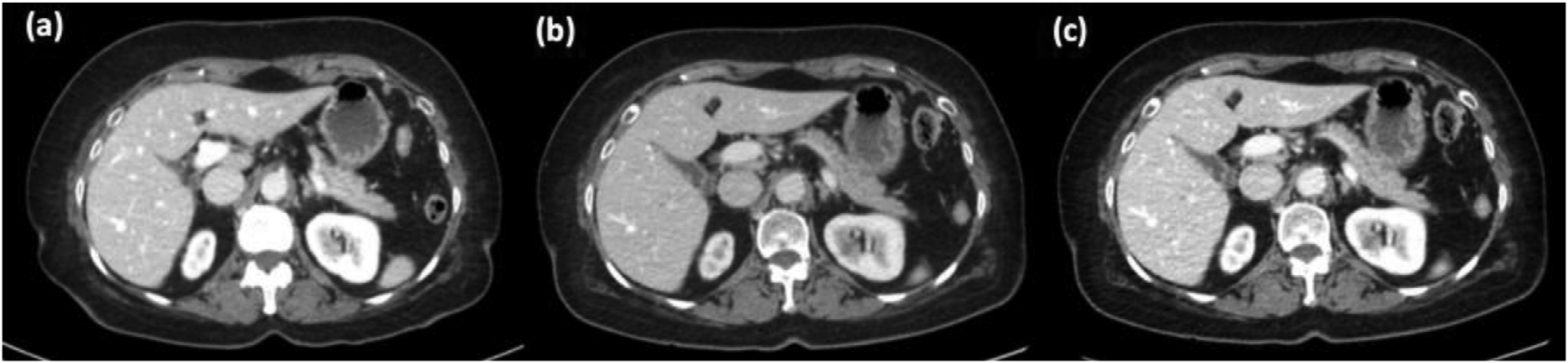
Cross sectional axial slices of the same patient with standard and then reduced ICM contrast dose. A 100 mm^2^ region of interest placed within the lumen of the descending aorta was used to measure the average CT number. (a) Full ICM dose displayed at 70 keV had an average HU measured in the aorta of 212.1 ± 12.1 HU. (b) Reduced ICM dose displayed at 70 keV had a CT number measured in the aorta of 156.4 ± 15.2 HU. (c) Reduced ICM dose displayed at 61 keV. The CT number measured in the aorta is 211.1 ± 19.1 HU. The window width and window level are set to 400/40 HU for all images.

The first patient was a 70‐year‐old female, 62 kg that measured an effective diameter of 26.3 cm. The patient's DECT Urogram was performed with a standard ICM dose protocol and images were processed as a 70 keV VMI (Figure 7(a)). On a follow up examination, 4 months later (effective diameter 26.9 cm), the patient received a DECT Urogram with a reduced ICM dose protocol; reconstructed images included a 70 keV (Figure 7(b)) and a 61 keV (Figure 7(c)), VMI. Full and reduced ICM contrast at 70 keV and 61 keV VMI qualitatively appeared to have similar vascular enhancement. Empirically, the 70 keV full ICM dose measured 212.1 ± 12.1 HU, and the reduced ICM dose aorta at 61 keV measured 211.1 ± 19.1 HU, a 0.5% difference. Using psoas muscle region to obtain background CT number and noise, the calculated CNR is 9.82 (70 keV with full ICM dose), 2.89 (70 keV with reduced ICM dose), and 8.33 (61 keV with reduced ICM dose), respectively.

**FIGURE 8:**
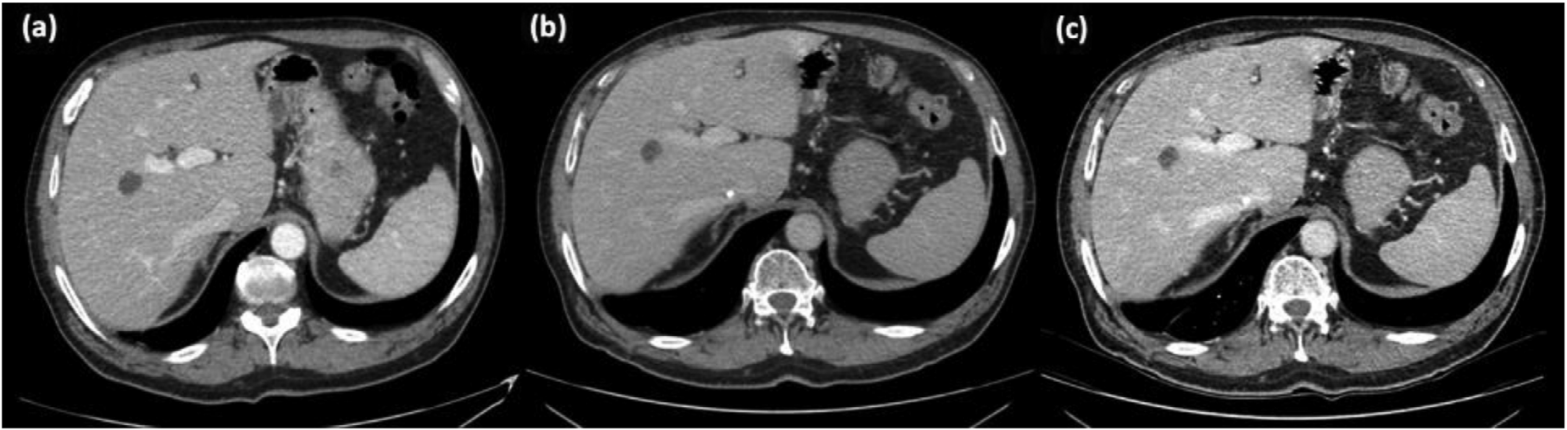
Cross sectional axial slices of the same patient with standard and then reduced ICM contrast dose. (a) Full ICM dose displayed at 70 keV had a CT number measured in the descending aorta of 215.6 ± 17.4 HU. (b) Reduced ICM dose displayed at 70 keV had a CT number measured in the descending aorta of 100.1 ± 18.1 HU. The window width (WW)/window level (WL) was set to 400/40 HU. (c) Reduced ICM dose displayed at 45 keV. The CT number measured in the descending aorta is 218.1 ± 37.3 HU. The WW/WL was set to 536/30 HU.

The second patient was a 68‐year‐old male, 80 kg that measured an effective diameter of 31 cm. DECT Urogram was performed with a standard ICM dose protocol and images were processed as a 70 keV VMI (Figure 8(a)). On a follow up examination 4 months later (effective diameter 31.1 cm), the patient underwent DECT Urogram with a reduced ICM dose protocol; reconstructed images included a 70 keV (Figure 8(b)) and a 45 keV (Figure 8(c)), VMI. Full ICM contrast reconstructed at 70 keV and reduced iodine ICM contrast at 45 keV qualitatively appeared to have similar hepatic vascular enhancement. Empirically measured CT number values from the descending aorta demonstrated that the 70 keV full ICM dose protocol (*215.6 ± 17.4 HU)*, and the reduced ICM dose protocol, at 45 keV (*218.1 ± 37.3 HU)*, measured a 1.1% difference. CNR was 10.19 (70 keV with full ICM dose), 3.39 (70 keV with reduced ICM dose), and 4.82 (45 keV with reduced ICM dose), respectively.

In both clinical examples, patients receiving a reduced ICM contrast dose, when the VMIs were processed at 70 keV, presented with qualitatively lower vascular contrast and measured 26% (Figure 7(b)), and 54% (Figure 8(b)), lower CT number values for patients 1 and 2, respectively. Although confounding factors, such as patient condition, human physiology and the physics of CT imaging (i.e., beam hardening) influence the aorta CT number values, the observed decrease is primarily attributed to the 33.3% reduction of iodinated contrast material.

## 4 DISCUSSION

In this study, CT attenuation and CNR values of ICM contrast inserts were imaged in different sized pediatric and adult physical anthropomorphic phantoms to identify the achievable ICM dose reduction for SECT and DECT scanners. For SECT scanners, the kV station and reconstruction algorithms (i.e., SBIR or DLR) were adjusted to optimize CNR. For DECT scanners, CNR was optimized using VMI energy levels. The results demonstrated ICM dose reduction potential for SECT scanners was on the order of 21%–58% for 100 and 80 kV images reconstructed with SBIR, respectively, and 27%–80% for DLR images. For DECT, achievable ICM contrast reduction levels were dependent on scanner DECT type and phantom sizes, but ranged from an average of 15% at 68/70 keV to 74% at 40 keV, when compared to full ICM contrast at 120 kV.

Our findings suggest that an ICM dose reduction is possible when scanning with DECT and reconstructing lower energy VMI's. Although reducing tube potential with SECT can also achieve contrast dose reduction while maintaining CNR, DECT offers additional options and advantages in this regard. The feasibility of ICM contrast reduction using DECT has been demonstrated in clinical studies. Tsang et al. reported comparable or better CNR was achieved on DECT derived 50 keV images with 50% reduction in contrast dose, in comparison to single‐energy 120 kV images in contrast enhanced pediatric CT exams.^14^ Carrascosa et al. reported comparable interpretability of coronary angiography between DECT derived 60 keV images acquired with half iodine dose and single energy 100 or 120 kV images.^15^ In a multiphasic hepatic CT study, Nagayama et al. showed that with 50% of iodine contrast dose, greater CNR was achieved at DECT derived 40–50 keV images than single energy 120 kV images.^16^ The strength of our study is that we provided a baseline or idealized estimate of the magnitude of iodine contrast reduction while maintaining CNR between SECT images and VMIs from three different major DECT platforms across a range of phantom sizes.

The plots in Figures 3, 4, 5, 6 and Equations (5)–(6) can help institutions realize the full potential of their scanner technology. Although we indicate that a contrast reduction of upwards of 74%–80% is achievable with a single vendor without any loss of CNR, one must consider several factors, such as the diagnostic task, post‐injection scan start time, quality of the bolus, anatomical noise, and patient physiology. Also, the results are based on phantom data where scan conditions can be controlled, unlike the clinical reality faced in various medical centers. Hence, the percent reductions we denote are an idealized lower bound, after which the CNR will start to deteriorate in the phantoms used in this study; thus, the percent contrast reduction without any loss of CNR in patients of varying sizes and body characteristics will likely be less.

Nevertheless, this study highlights the importance of comprehensively identifying the limitations of a given scanner and the need to approach any ICM dose reduction effort in an iterative step‐wise approach that includes comparisons with prior exams, modeling the capability of a given scanner, and, if possible, involving multiple radiologists to perform a reader study. In addition, modern‐day radiology has incorporated several artificially intelligent radiological systems. Many of these AI systems were trained with retrospective, full‐contrast dose scans. Since the noise magnitude, texture, and uptake patterns in tissues of reduced contrast scans may differ, the predictions of AI systems should be closely scrutinized for inaccuracy when processing reduced‐contrast scans.

Additionally, the pediatric radiology community has received less attention for DECT applications because of (1) a lack of evidence‐based data demonstrating improved clinical outcomes and (2) some of the current DECT vendors do not provide pediatric‐specific scanning protocols. The attraction of DECT is that it delineates and quantifies material composition by scanning with low and high‐energy polychromatic spectra. The perception of noise is reduced in higher energy VMIs, while the lower energy VMIs, closer to the K‐edge of iodine (*z* = 53, K‐edge = 33.2 keV), improve the overall contrast of the image at the cost of higher noise. With DECT, the optimal CNR for target structures is often found around a VMI of 70 keV, but with model‐based iterative reconstruction or deep learning image reconstruction, optimal CNR value could be achieved at VMIs of 40–50 keV.^17^, ^18^, ^19^ Since the lower VMIs enhance the conspicuity of iodinated contrast, the amount of contrast required to achieve diagnostically acceptable exams can be reduced while lowering the VMI keV.

There are several limitations of our study. First, we did not assess the potential of material decomposition iodine images. Several literature reports have suggested the potential value of DECT‐derived material specific images.^20^, ^21^, ^22^ These images may further improve the conspicuity of contrast‐enhanced structures and should be considered in any clinical routine that may use DECT. Second, only CNR was used as the image quality metric for our optimization scheme. The shifts in noise texture or resolution due to IR,^23^ DLR, or DECT material decomposition were not considered. Furthermore, although CNR is found to be universally higher at lower kV (SECT) or keV (DECT), images of lower kV or keV are usually more suspectable to image artifacts, such as increased noise and beam hardening artifact. Hence, these additional image quality metrics should be evaluated when adopting the contrast medium reduction strategy in patient exams. The presented optimization scheme is also constrained by radiation dose. Depending on available resources and during shortages of drugs, it may be worth considering the merits of an increase in radiation dose to reduce image noise and allow for even lower administered contrast dosages. Third, the applicability of our results is limited in two aspects: 1) the range of phantom sizes were different in the low SECT kV and DECT versus SECT sections, respectively. 2) Two SECT and three DECT systems were included in the two sections. Fourth, this study employed solid contrast rods of iodine and may represent diagnostic tasks more aligned with solid organ perfusing and vascular enhancing imaging tasks. The results from this study may not hold for other tasks such as assessment of iodine enhancing lesions or delayed nephrographic scans.

## 5 CONCLUSIONS

In this study, we compared CNR for different concentrations of iodinated contrast rods within phantoms of various pediatric and adult sizes and used the method to determine the contrast‐dose reduction capability of different SECT and DECT vendors and scan parameters. ICM dose reduction for SECT scanners, with CNR matched at 120 kV, ranged from 21% to 80%, where DLR did not provide enhanced ICM reduction for scanner A but allowed for an additional 40% ICM reduction for scanner B. For DECT scanners with CNR matched at 120 kV, average ICM reductions over all phantoms ranged for: Fast‐kV switch—15% to 35% with VMIs between 40 and 68 keV, dual‐source—22% to 52% with VMIs between 40 and 80 keV, and dual‐layer—22% and 74% with VMIs between 40 and 70 keV; similar trends of ICM reductions were found when matching CNR between VMIs and 100 kV images.

## AUTHOR CONTRIBUTIONS

Study concepts/study design and data analysis/interpretation: all authors; manuscript drafting and manuscript revision: all authors; final approval of the submitted manuscript: all authors; agreement to be accountable for all aspects of the work in ensuring that questions related to the accuracy or integrity of any part of the work are appropriately investigated and resolved: all authors; literature research: Jia Wang, Usman Mahmood, Sarah McKenney, Samuel Brady, experimental studies: Jia Wang, Xinhui Duan, Usman Mahmood, Samuel Brady.

## CONFLICT OF INTEREST STATEMENT

The authors have no relevant conflicts of interest to disclose.
